# The association between cigarette smoking and health care service utilization among middle-aged and elderly adults in China

**DOI:** 10.3389/fpubh.2022.952357

**Published:** 2022-11-02

**Authors:** Jiarui Song, Chenggang Jin, Xi Cheng

**Affiliations:** ^1^School of Social Development and Public Policy, Beijing Normal University, Beijing, China; ^2^Research Center for Health and Social Policy, Beijing Normal University, Zhuhai, China

**Keywords:** China, health care utilization, middle-aged, smoking index, tobacco use

## Abstract

**Objective:**

To assess the associations between tobacco use and health care service utilization in Chinese individuals aged more or equal to 40 years old.

**Method:**

This research was a cross-sectional study using data from eight provinces in China, and the final sample consisted of 4,733 observations (4,749 participants) aged more or equal to 40 years old. The dependent variable was health care utilization measured by outpatient and inpatient service utilization. Descriptive statistics were used to summarize the socio-demographic characteristics of the sample according to smoking status. The association between tobacco use and health care service utilization was examined by an instrumental variable (IV) probit model.

**Results:**

Of the respondents interviewed in 2020, 3,116 (65.84%) were never smokers, 654 (13.82%) were smokers with the smoking index (SI) <400, and 963(20.34%) were smokers with SI≥400. Smokers with SI <400 reported a 6.80% higher probability of using outpatient services. Smokers with SI <400 and SI≥400 reported a 3.10 and 4.20% higher average probability of using ≥3 outpatient visits than never smokers, respectively. Additionally, smokers with SI <400 and SI≥400 reported a 6.30 and 6.20% higher average probability of using inpatient services than those who had not smoked. Moreover, smokers with SI≥400 were more likely to have had ≥2 hospital visits than nonsmokers.

**Conclusions:**

Smokers make greater use of health care services. Control of smoking may ease the burden of related health care utilization.

## Introduction

The tobacco epidemic has become a public health problem around the world that endangers public health and causes severe health and economic consequences. Tobacco was responsible for 8.71 million deaths in 2019. In addition, the global burden of disease study has shown that if tobacco consumption could be stopped, it could have potentially prevented 15% of the deaths that occurred globally in 2019 (1). Furthermore, the economic burden related to tobacco is rising. Goodchild et al. demonstrated that the direct economic cost of smoking-attributable diseases in 2012 accounted for 5.7% of global health expenditures (2).

China is the largest manufacturer and consumer of cigarettes worldwide (3). A study investigated the prevalence of tobacco smoking among Chinese, and their results showed that 62.4% of Chinese adult men reported histories of smoking in 2010; specifically, 54% of them were current smokers, and only one-third of these current smokers intended to quit (4). Tobacco consumption results in approximately one million deaths in China (5). Moreover, the economic burden of smoking-attributable diseases worldwide is substantial, and it is the same in China. A systematic review of 20 studies related to the economic burden of smoking-attributable disease in China indicated that the estimated total cost of smoking ranged from 57 to 368 billion RMB; specifically, outpatient visits accounted for up to 98% of the direct costs (6). Moreover, Yang et al. performed a pooled meta-analysis to explore the trend of tobacco smoking in various regions and showed that the smoking prevalence rate for male ever-smokers continues to increase in China; in addition, they reported that current smoking was associated with the risk of death (7). In general, these findings indicate that China is facing a dilemma of sustained increases in the health burden of tobacco.

The fast aging population and the increased prevalence of chronic diseases are current health care challenges China faces (8–12). Smoking is a preventable risk factor for various diseases, including cardiovascular disease, lung cancer, diabetes, and chronic respiratory disease (13–15). Additionally, smoking accelerated the epidemic of noncommunicable chronic diseases in China (16–18). Therefore, to reduce the tobacco-attributable disease burden and allocate health service resources effectively, it is necessary to assess the effect of smoking on health care utilization.

Various studies have investigated the impact of tobacco consumption on the use of outpatient services. Kahende et al. explored smoking status differences in health care utilization from a large population. They found that current and former smokers who had quit for < 2 years were more likely to have outpatient visits than never smokers after controlling for various factors (19). This finding was also reported by Li et al. using a Chinese population (20). On the other hand, a study from Azagba et al. reported conflicting results. The authors divided participants into two groups—low and high users; they found no association between higher utilization of general practitioners, specialists, and current smokers in the group of low users, and current smokers had fewer hospital visits than never smokers (21). In addition, many studies have reported the effect of smoking on inpatient care utilization. Researchers have found that, current and former smokers are more likely to use hospitalization services than people who have never smoked; additionally, smoking is strongly associated with increased use of hospital services (19, 21–23). Although China is the largest manufacturer and consumer of cigarettes, there are few studies estimated the impact of cigarette smoking on health care utilization among the Chinese population. In addition, it is necessary to focus on middle-aged people because there is evidence that smoking's cumulative influence on health can impact middle-aged people more for the utilization of health care services due to smoking-attributable disease than young people (24). In addition, previous studies have demonstrated methodological limitations, especially in cross-sectional data, and studies have commonly reported endogeneity problems. Additionally, whether to become a smoker is the result of individual self-selection. Endogeneity problems caused by omitted variables may result in a biased estimation. Hence, to control for endogeneity, the instrumental variable (IV) technique is commonly used (25, 26).

Therefore, the present study aimed to assess the associations between tobacco use and health care service utilization among Chinese individuals aged more or equal to 40 years old using an instrumental variable (IV) probit model. We hypothesize that smoking is positively associated with health care service utilization.

## Materials and methods

### Study design

We conducted a household survey from November 1, 2019, to January 30, 2020. Participants were Chinese individuals aged more or equal to 40 years old from 24 primary health care facilities (PHC). A stratified multi-stage sampling method was taken. Firstly, we selected eight province-level regions (Hebei, Heilongjiang, Shandong, Henan, Shanxi, Hubei, Sichuan, Guizhou) according to the level of economic development. Then, we randomly selected 2–4 PHC institutions in each province as our investigation units. Approximately 100 households of each PHC were chosen randomly. The inclusion criteria were as follows: the head of the household has a minimum of 40 years, lived in the area for at least half a year, and was willing to participate in the study. For observational studies involving logistic regression in the analysis, a minimum of 500 sample size is recommended to derive statistics that represent the parameters of the target population (27). Therefore, with eight provinces selected in the research, this observational study needed at least 4,000 participants. After eliminating 16 missing data about family income, the final sample consisted of 4,733 observations aged more or equal to 40 years old (4,749 participants).

In this study, participants had face-to-face interviews with data collectors, who had received adequate training to ensure the reliability of the survey. They used validated questionnaires that includes general household information, basic demographics, smoking behavior, health status, health care utilization and health insurance, and household economy. The Cronbach's alpha of the questionnaire was 0.775. There was a contact person in each investigation unit who was under the leadership of the research team. The contact person checked all the questionnaires before handing over to the research team. Then, the research team double-checked all the questionnaires and made phone call to participants if necessary. We obtained informed consent from all the participants prior to the enrollment. This study received approval from the ethics committee of the Capital Institute of Pediatrics, Beijing (ID: SHERLL2020017).

### Measures

#### Dependent variables

The study measured health care utilization by outpatient and inpatient service utilization. We obtained data by asking “How many times have you seen a doctor during the past year,” and “How many times have you been hospitalized during the past year.” Outpatient service utilization and inpatient service utilization were binary variables coded 1 if participants have used health service at least once and 0 otherwise. Moreover, the number of outpatient visits and hospital admissions were grouped into two levels. This cut-off point was chosen because approximately half of the participants had ≥3 outpatient visits and ≥2 hospitalizations. Details of the definition of dependent variables were shown in Table 1.

**Table 1:** The definition and abbreviation of selected variables in the study.

**Variable**	**Abbreviation**	**Definition**
Smoking index	SI	Categorical variable scored 1 if 0 < SI < 400, 2 if SI≥400, and 0 if respondents were never smokers.
Outpatient service utilization	Outpatient	Binary variable scored 1 if respondents had outpatient visits in the past year and 0 otherwise
Number of outpatient visits ≥3	-	Binary variable scored 1 if respondents had outpatient visits ≥3 times in the past year and 0 if respondents had an outpatient visit
Inpatient service utilization	Inpatient	Binary variable scored 1 if respondents had hospital admissions in the past year and 0 otherwise
Number of times hospitalized ≥2	-	Binary variable scored 1 if respondents had hospital admissions ≥2 times in the past year and 0 if respondents had a hospital admission
Age	Age	Continuous variable measured in years
Gender	Gender	Binary variable scored 1 for males and 0 for females
Educational level	Education	Categorical variable scored 1 for Illiterate, 2 for Primary School, 3 for Middle School, 4 for High School and Junior College, and 5 for College and above
Marital status	Marriage	Categorical variable scored 1 for Single, 2 for Married, and 3 for Divorced or Widowed
Logarithm of family income	Lnincome	Continuous variable
Residence location	Residence	Binary variable scored 1 for rural and 2 for urban
Work status	Work	Binary variable scored 1 for employed people, 0 for Unemployed people
Health insurance	Insurance	Binary variable scored 1 for insurance covered, 0 for no insurance covered
Health status	Health status	Binary variable scored 1 for at least one chronic disease, 0 for no chronic diseases.
Drink	Drink	Binary variable scored 1 if respondents had drinking habits and 0 otherwise
Whether the increase in the price of cigarettes reduced smoking	PRS	Binary variable scored 1 if the increase in cigarette prices reduced the number of cigarettes smoked and 0 otherwise

#### Independent variables

In the current study, we focused on cigarette smoking. Respondents were asked “Do you smoke now?.” The corresponding options were: (1) No, (2) Yes, and (3) Have quit smoking. Respondents who answered option (1) were classified as never smokers. Respondents who reported option (2) or (3) were asked additional questions about smoking history and amount. The smoking index (SI) was adopted and was defined as the root number of cigarettes smoked every day multiplied by the smoking years (28). Then, the cigarette smoking was categorized as never smokers, SI <400, and SI≥400.

#### Covariates

The covariates included economic status (logarithm of household income), medical insurance schemes, individual characteristics (gender, age, marital status, educational level, working status, residence location), drinking habits, and health status (hypertension, diabetes, chronic obstructive pulmonary disease, coronary heart disease, stroke, and cancer). Definitions of all relevant variables are provided in Table 1.

#### Instrumental variable

We used whether the increase in cigarette prices reduced smoking as an instrumental variable (29).

### Data analysis

The challenge of this study is to overcome endogeneity problems existed in observational studies and we adopted an instrument variable (IV) probit model to control for potential endogeneity problems. The instrumental variable was whether the increase in the price of cigarettes reduced smoking. It was associated with smoking behavior but did not directly affect health care service utilization, therefore fulfilling the instrumental variable exogeneity requirement. The IV probit model was adjusted for gender, age, marital status, education, working status, economic status, medical insurance schemes, health status, and drinking habits.

To further estimate the average treatment effect on the treated (ATET) of smoking on health care service utilization, we performed marginal analysis using the parameter estimates from the IV probit model. ATET is the estimated average difference of the treatment and control potential outcomes in the treated population. It can help obtain interpretable effects when the coefficient estimates are not directly useful.

The IV probit model is constructed as follows:


(1) $$utilization_{i}\;=\;\{\begin{array}{c} 1\;if,\;utilization_{i}^{*}>0\\ \;\\ 0\;if,\;otherwise\; \end{array}$$

(2) $$\begin{array}{l} utilization_{i}^{*}\;=\;\beta_{1}Smoke_{i}\;+\;\gamma Z\;+\;\mu_{i} \end{array}$$

(3) $$\begin{array}{l} Smoke_{i}\;=\;\pi_{1}Z\;+\;\pi_{2}I\;+\;\alpha_{i} \end{array}$$

*utilization*_*i*_ refers to the health care service utilization of the respondents. $utilization_{i}^{*}$ is the latent variable of health care service utilization in Equation (1). *Smoke*_*i*_, the independent variable of interest. β_1_ is the coefficient of interest. It captures the estimated effect of smoking on health care service utilization. *Z* is a rich set of individual characteristics; *I* is the instrumental variable; γ, π_1_ and π_2_ are the vectors of parameters for the control variables; μ_*i*_ and α_*i*_ are the error terms in the equation; and *i* denotes an individual respondent.

All analyses were performed by Stata 17.0 (Stata Corp, College Station, TX, USA), and the statistical significance was set at P < 0.05.

## Results

### Sample characteristics

Table 2 shows the distribution of demographic characteristics of never smokers, SI <400, and SI≥400. Of the respondents interviewed in 2020, 3,116 (65.84%) were never smokers, 654 (13.82%) were smokers with SI <400, and 963(20.34%) were smokers with SI≥400. The average ages of SI <400 and SI≥400 were 57.55 ± 13.01 and 57.34 ± 10.51, respectively. Never smokers tended to be younger than smokers. Never smokers (43.66%) included a higher proportion of males than smokers with SI≥400 (37.44%) and smokers with SI <400 (18.90%). There were significant differences in marital status (*p* < 0.001), educational status (*p* < 0.001), work status (*p* < 0.001), residence location (*p* < 0.001), health status (*p* < 0.001), and drinking habits (*p* < 0.001) while the logarithm of family income (*p* = 0.520) and health insurance (*p* = 0.626) were not significant.

**Table 2:** Description of the selected samples of Chinese adults ≥40 years old by smoking status (*N* = 4,733).

**Characteristics**	**Smoking status**			***p*-value**
	**Never smoked**	**SI < 400**	**SI≥400**	
	**N(%)/Mean**	**N(%)/Mean**	**N(%)/Mean**	
Age	55.26 ± 11.02	57.55 ± 13.01	57.34 ± 10.51	< 0.001
Gender				< 0.001
Male	1,067 (43.66)	462 (18.90)	915 (37.44)	
Female	2,049 (89.52)	192 (8.39)	48 (2.09)	
Marital status				< 0.001
Single	52 (45.61)	21 (18.42)	41 (35.97)	
Married	2,767 (66.85)	538 (13.00)	834 (20.15)	
Divorced/Widowed	297 (61.88)	95 (19.79)	88 (18.33)	
Education				< 0.001
Illiterate	419 (66.61)	115 (18.28)	95 (15.11)	
Primary school	748 (62.86)	158 (13.28)	284 (23.86)	
Middle school	1,052 (66.12)	176 (11.06)	363 (22.82)	
High school/junior college	500 (65.96)	112 (14.77)	146 (19.27)	
University/college and above	374 (69.91)	89 (16.64)	72 (13.45)	
Work				< 0.001
No	1,007 (68.36)	241 (16.36)	225 (15.27)	
Yes	2,109 (64.69)	413 (12.67)	738 (22.64)	
Lnincome	10.79	10.64	10.50	0.520
Residence location				< 0.001
Rural	2,060 (64.47)	423 (13.24)	712 (22.29)	
Urban	1,056 (68.67)	231 (15.02)	251 (16.31)	
Health insurance				0.626
No	65 (67.71)	15 (15.63)	16 (16.66)	
Yes	3,051 (65.79)	639 (13.78)	947 (20.42)	
Health status				< 0.001
0	2,231 (70.09)	349 (10.96)	603 (18.95)	
1	885 (57.09)	305 (19.67)	360 (23.24)	
Drink				< 0.001
No	2,512 (76.17)	363 (11.01)	423 (12.29)	
Yes	604 (42.09)	291 (20.28)	540 (37.63)	
Total	3,116 (65.84)	654 (13.82)	963 (20.34)	

### The association between smoking and health care service utilization

Columns (i) and (iii) of Table 3 present the results of the probit regression model (outpatient care utilization). After controlling for smoking index, age, sex, marital status, education, income, residence, health insurance, health status, and drinking habits, smokers with SI <400 increased the probability of having an outpatient visit by 18.50% (*p* < 0.001). Smoking was positively related to the number of outpatient visits, while the results are not significant (*p* > 0.05).

**Table 3:** Probit regression analysis and IV probit regression analysis of outpatient service utilization.

	**Outpatient service utilization**			
	**Visit (Y/N)**		**Number of outpatient visits (**≥**3 times)**	
	**Probit**	**IV Probit**	**Probit**	**IV Probit**
	**(i)**	**(ii)**	**(iii)**	**(iv)**
Smoking index (reference group: Never smoked)
SI < 400	0.185** (0.060)	0.165** (0.069)	0.110 (0.084)	0.221* (0.095)
SI≥400	0.107 (0.056)	0.091 (0.089)	0.075 (0.085)	0.272* (0.127)
Age	0.008*** (0.002)	0.008*** (0.002)	0.017*** (0.003)	0.017*** (0.003)
Gender (reference group: Female)			
Male	0.025 (0.047)	0.044 (0.046)	0.172** (0.071)	0.189** (0.072)
Education level (reference group: Illiterate)
Primary school	−0.051 (0.067)	−0.081 (0.067)	0.043 (0.075)	−0.008 (0.089)
Middle school	−0.084 (0.069)	−0.159 (0.068)	−0.069 (0.080)	−0.016 (0.095)
High school/junior college	−0.133 (0.081)	−0.319 (0.078)	−0.125 (0.098)	0.058 (0.118)
University/college and above	−0.150 (0.097)	−0.447 (0.090)	−0.203 (0.127)	0.071 (0.147)
Marital status (reference group: Single)
Married	−0.171 (0.130)	−0.154 (0.126)	0.026 (0.155)	−0.023 (0.175)
Divorced/widowed	−0.003 (0.143)	−0.051 (0.139)	−0.025 (0.168)	−0.153 (0.191)
Residence location (reference group: Rural)
Urban	−0.376*** (0.048)	−0.255*** (0.049)	−0.027 (0.062)	−0.033 (0.072)
Lnincome	−0.019 (0.023)	−0.025 (0.023)	−0.042 (0.028)	−0.016 (0.029)
Work (reference group: No)
Yes	0.047* (0.045)	0.042 (0.046)	−0.074 (0.063)	−0.073 (0.065)
Health insurance (reference group: No insurance)
Insurance	−0.257 (0.137)	−0.255 (0.132)	−0.232*** (0.054)	−0.518*** (0.180)
Health status (reference group: No chronic disease)
1	0.652*** (0.044)	0.652*** (0.044)	0.738*** (0.051)	0.514*** (0.063)
Drink (reference group: no drink)
Drink	−0.123* (0.047)	−0.122* (0.047)	−0.083 (0.060)	0.028 (0.072)
_cons	−0.031 (0.324)	−0.041 (0.329)	−0.776 (0.492)	−0.842* (0.441)
*N*	4,733	4,733	4,733	4,733

Standard errors in parentheses. *p < 0.05, **p < 0.01, ***p < 0.001.

Column (ii) and (iv) of Table 3 report the estimated results of the IV probit regression model. The estimated coefficient reported positive and significant associations between smoking and the number of outpatient visits (*p* < 0.05).

As shown in **Table 5**, the ATET of 0.068 shows that for smokers with SI <400, the average probability of outpatient utilization would be 6.80% higher than if they had not smoked (*p* < 0.01). The ATET of 0.031 and 0.042 indicates that for smokers with SI <400 and SI≥400, the average probability of having higher outpatient service utilization would be 3.10% (*p* < 0.05) and 4.20% (*p* < 0.05), respectively higher than if they had not smoked.

Columns (i) and (iii) of Table 4 present the results of the probit regression model (inpatient care utilization). After controlling for smoking index, age, sex, marital status, education, income, residence, health insurance, health status, and drinking habits, smokers with SI <400 and SI≥400 increased the probability of having a hospital admission by 22.10% (*p* < 0.001) and 17.50% (*p* < 0.001), respectively. Smokers with SI≥400 were positively associated with the number of hospitalizations (*p* < 0.05).

**Table 4:** Probit regression analysis and IV probit regression analysis of inpatient service utilization.

	**Inpatient service utilization**			
	**Hospitalization (Y/N)**		**Number of times hospitalized (**≥**2 Times)**	
	**Probit**	**IV Probit**	**Probit**	**IV Probit**
	**(i)**	**(ii)**	**(iii)**	**(iv)**
Smoking index (reference group: Never smoked)
SI < 400	0.221*** (0.070)	0.267*** (0.080)	0.075 (0.093)	0.143 (0.110)
SI≥400	0.175*** (0.069)	0.263* (0.103)	0.190* (0.094)	0.309* (0.141)
Age	0.012*** (0.002)	0.012*** (0.003)	0.008*** (0.004)	0.009*** (0.003)
Gender (reference group: Female)			
Male	−0.007 (0.060)	−0.001 (0.061)	0.015 (0.081)	0.031 (0.082)
Education level (reference group: Illiterate)
Primary school	0.043 (0.075)	0.043 (0.076)	−0.061 (0.096)	−0.049 (0.092)
Middle school	−0.069 (0.080)	−0.066 (0.081)	−0.144 (0.104)	−0.117 (0.100)
High school/junior college	−0.125 (0.098)	−0.124 (0.100)	−0.229 (0.133)	−0.175 (0.125)
University/college and above	−0.203 (0.126)	−0.198 (0.127)	−0.187 (0.169)	−0.092 (0.157)
Marital status (reference group: Single)
Married	0.025 (0.155)	0.023 (0.153)	0.257 (0.222)	0.244 (0.208)
Divorced/widowed	−0.025 (0.168)	−0.025 (0.168)	0.124 (0.236)	0.123 (0.229)
Residence location (reference group: Rural)
Urban	−0.027 (0.062)	−0.028 (0.062)	0.113 (0.083)	0.112 (0.079)
Lnincome	−0.042 (0.028)	−0.039 (0.028)	−0.078* (0.037)	−0.076* (0.037)
Work (reference group: No)
Yes	−0.232*** (0.054)	−0.232*** (0.054)	−0.267*** (0.072)	−0.268*** (0.070)
Health insurance (reference group: No insurance)
Insurance	0.145 (0.193)	0.145 (0.211)	0.007 (0.262)	0.006 (0.301)
Health status(reference group: No chronic disease)
1	0.738*** (0.052)	0.738*** (0.051)	0.820*** (0.072)	0.819*** (0.067)
Drink (reference group: no drink)
Drink	−0.083 (0.060)	−0.081 (0.061)	−0.167* (0.084)	−0.167* (0.083)
_cons	−1.594*** (0.411)	−1.649*** (0.415)	−1.69** (0.548)	−1.76*** (0.555)
*N*	4,733	4,733	4,733	4,733

Standard errors in parentheses. *p < 0.05, **p < 0.01, ***p < 0.001.

Columns (ii) and (iv) of Table 4 present the estimated results of the IV probit regression model. The estimated coefficient results show that smoking positively affects hospital admissions, and smokers with SI≥400 were related to the number of times hospitalized.

As shown in Table 5, the estimated ATET implies that smokers reported approximately 6.20% higher average probabilities of using inpatient services than never smokers. The ATET of 0.051 shows that for smokers with SI≥400, the average probability of higher hospital admissions increased by 5.10% when SI≥400 vs. never smokers (*p* < 0.05).

**Table 5:** Estimated ATET of smoking on outpatient service utilization and inpatient service utilization.

	**Margin**	**Unconditional Std. Err**.	**z**	**P>z**	**[95% Cl]**	
ATET
Outpatient service utilization (Y/N)				
(SI < 400 vs. never smoked)	0.068	0.023	2.87	0.004	0.021	0.115
(SI≥400 vs. never smoked)	0.043	0.031	1.38	0.169	−0.018	0.104
Number of outpatient visits (≥3 Times)
(SI < 400 vs. never smoked)	0.031	0.015	2.11	0.035	0.002	0.059
(SI≥400 vs. never smoked)	0.042	0.028	2.00	0.046	0.000	0.082
Inpatient service utilization (Y/N)
(SI < 400 vs. never smoked)	0.063	0.019	3.21	0.001	0.024	0.100
(SI≥400 vs. never smoked)	0.062	0.025	2.45	0.014	0.012	0.110
Number of inpatient visits (≥2 Times)
(SI < 400 vs. never smoked)	0.017	0.013	1.26	0.207	−0.009	0.044
(SI≥400 vs. never smoked)	0.051	0.020	1.99	0.046	0.000	0.080

In Table 3, the coefficient for the age indicates that the likelihood of using outpatient services and the number of outpatient visits increase with age (*p* < 0.001). Participants who lived in cities (*p* < 0.001) and had drinking habits (*p* < 0.05) were less likely to have an outpatient visit. Male sex was positively related to increased outpatient visits (*p* < 0.01). The impact of health insurance on increased outpatient visits were negative but significant (*p* < 0.001). Health status was positively associated with outpatient services (*p* < 0.001).

In Table 4, the coefficient for the age indicates that the likelihood of using inpatient services and the number of hospital visits increase with age (*p* < 0.001). Family income (*p* < 0.05) and drinking habits (*p* < 0.05) were negatively associated with the frequency of hospital visits. Health status was positively associated with inpatient services (*p* < 0.001).

Results from Table 6 indicate that, people between the ages of 40 and 49 with SI <400 were 25.70% (*p* < 0.05) more likely to have outpatient services than those who never smoke. People between the ages of 50 and 59 with SI <400 and SI≥400 were 51.90% (*p* < 0.001) and 26.30% (*p* < 0.05) more likely to be hospitalized than never smokers, respectively.

**Table 6:** Effect of smoking on outpatient service utilization and inpatient service utilization among different age groups.

	***N* in sample**	**Outpatient service utilization (Y/N)**	**Inpatient service utilization (Y/N)**
40–49 years	1,663		
Never smoked	1,185 (reference)		
SI < 400	228	0.257* (0.105)	0.070 (0.169)
SI≥400	250	0.092 (0.107)	0.259 (0.165)
50–59 years	1,441		
Never smoked	907 (reference)		
SI < 400	171	0.203 (0.116)	0.519*** (0.135)
SI≥400	333	0.015 (0.100)	0.263* (0.130)
60+ years	1,659		
Never smoked	1,024 (reference)		
SI < 400	225	0.115 (0.097)	0.173 (0.097)
SI≥400	380	0.149 (0.089)	0.105 (0.096)

Standard errors in parentheses. *p < 0.05, **p < 0.01, ***p < 0.001. All the models were adjusted for economic status, medical insurance schemes, individual characteristics, drinking habits, and health status.

## Discussion

Using an IV probit model, we assess the associations between tobacco use and health care service utilization among Chinese individuals aged more or equal to 40 years old. The results support our hypothesis that smoking is positively associated with the utilization of health care services. Specifically, smokers with SI <400 reported a 6.80% higher probability of using outpatient services. Smokers with SI <400 and SI≥400 reported a 3.10 and 4.20% higher average probability of using ≥3 outpatient visits than never smokers. Additionally, smokers with SI <400 and SI≥400 reported a 6.30 and 6.20% higher average probability of using inpatient services than those who had not smoked. Moreover, participants with SI≥400 were more likely to have had ≥2 hospital visits than nonsmokers.

Our findings are consistent with previous studies in other countries, such as the USA (19), Canada (21), Spain (22), Switzerland (30), Denmark (31), Germany (32), South Korea (33) and the Middle East (34). Furthermore, a recent cohort study from Hubei Province in China suggested that smoking behaviors, such as quitting smoking early and consuming a small amount of cigarettes, would decrease health care utilization and medical costs (10). Apart from hospital admission and the number of hospital visits, research from Hvidtfeldt et al. (31) and Fassmer et al. (32) found that smokers had longer duration stays in the hospital than those who never smoked, which supports the conclusion that smoking elevates health care utilization.

Positive associations between tobacco consumption and health care utilization are biologically plausible, as smoking is considered an important factor for many diseases requiring health care utilization. Many studies have documented the adverse health impacts of smoking, including cardiovascular diseases, diabetes, cancers, and chronic respiratory diseases (13, 15, 35–37). These health risks can result in the use of health services.

We also found that the impact of age on health care utilization may depend on different age groups. For example, the effect of age was positive and significant only in smokers aged 40–59 years, and the effect of age over 60 years was positive but not significant. Given that smoking is the leading cause of premature death in older people (38), there may be a healthy survivor effect in our sample that resulted in dilution of health services for older smokers.

Against the backdrop of an aging population and increasing health expenditures, the findings may have implications to help the government better reach the expected public health outcomes. To reduce the consumption of tobacco products and the tobacco-related disease burden, strongly enforced and comprehensive policies are urgently needed. For example, communities should carry out health education programs and highlight the health risks of smoking to smokers. In the meantime, when carrying out tobacco control activities, local health authorities should focus on middle-aged and elderly people and people with chronic diseases. Moreover, China still needs to continuously raise tobacco taxes and prices to reduce the tobacco consumption and mitigate the financial burden on the government. In addition, developing a tobacco excise tax and earmarking taxes for public health expenditure is necessary, as government spending on tobacco control is believed to be cost-effective.

In this study, the instrumental variable (IV) probit model overcomes the endogeneity problems that previous cross-sectional studies have faced. The estimated results for the IV probit regression model were mostly higher than those for the probit regression model, suggesting that the probit model may have underestimated the impact of smoking on health care use due to selection bias. However, the current study has some limitations that should be emphasized. First, self-reported data we used in the study may result in recall bias. Second, a cross-sectional design is unlikely to establish a causality between health care utilization and smoking behaviors. Longitudinal data is needed to further assess the cause-and-effect relationship between smoking and health care service utilization.

## Conclusion

The findings of this study showed that smoking elevates the use of health care services. This study could provide the government with better information on anti-smoking measures and inform health sector decisions on health care resource allocation. Interventions aiming to reduce smoking have the potential to greatly reduce health service utilization among middle-aged and older Chinese.

## Data availability statement

The data analyzed in this study is subject to the following licenses/restrictions: data involves personal privacy issues. Requests to access these datasets should be directed to JS, jrsong1993@163.com.

## Author contributions

JS made contributions to study design, developed the methodology, performed the statistical analysis, and wrote the manuscript. CJ developed the methodology and coordinated the analyses. XC developed the methodology, reviewed, and edited the manuscript. All authors contributed to the article and approved the submitted version.

## Conflict of interest

The authors declare that the research was conducted in the absence of any commercial or financial relationships that could be construed as a potential conflict of interest.

## Publisher's note

All claims expressed in this article are solely those of the authors and do not necessarily represent those of their affiliated organizations, or those of the publisher, the editors and the reviewers. Any product that may be evaluated in this article, or claim that may be made by its manufacturer, is not guaranteed or endorsed by the publisher.
